# Patient derived xenografts (PDX) predict an effective heparanase-based therapy for lung cancer

**DOI:** 10.18632/oncotarget.25022

**Published:** 2018-04-10

**Authors:** Amit Katz, Uri Barash, Ilanit Boyango, Sari Feld, Yaniv Zohar, Edward Hammond, Neta Ilan, Ran Kremer, Israel Vlodavsky

**Affiliations:** ^1^ Department of General Thoracic Surgery, Rambam Health Care Campus, Haifa, Israel; ^2^ Cancer and Vascular Biology Research Center, Rappaport Faculty of Medicine, Technion, Haifa, Israel; ^3^ Department of Pathology, Rambam Health Care Campus, Haifa, Israel; ^4^ Zucero Therapeutics, Brisbane, Queensland, Australia

**Keywords:** heparanase, lung cancer, PDX, PG545, metastasis

## Abstract

Heparanase, the sole heparan sulfate (HS) degrading endoglycosidase, regulates multiple biological activities that enhance tumor growth, metastasis, angiogenesis, and inflammation. Heparanase accomplishes this by degrading HS and thereby facilitating cell invasion and regulating the bioavailability of heparin-binding proteins. HS mimicking compounds that inhibit heparanase enzymatic activity were examined in numerous preclinical cancer models. While these studies utilized established tumor cell lines, the current study utilized, for the first time, patient-derived xenografts (PDX) which better resemble the behavior and drug responsiveness of a given cancer patient. We have previously shown that heparanase levels are substantially elevated in lung cancer, correlating with reduced patients survival. Applying patient-derived lung cancer xenografts and a potent inhibitor of heparanase enzymatic activity (PG545) we investigated the significance of heparanase in the pathogenesis of lung cancer. PG545 was highly effective in lung cancer PDX, inhibiting tumor growth in >85% of the cases. Importantly, we show that PG545 was highly effective in PDX that did not respond to conventional chemotherapy (cisplatin) and vice versa. Moreover, we show that spontaneous metastasis to lymph nodes is markedly inhibited by PG545 but not by cisplatin. These results reflect the variability among patients and strongly imply that PG545 can be applied for lung cancer therapy in a personalized manner where conventional chemotherapy fails, thus highlighting the potential benefits of developing anti-heparanase treatment modalities for oncology.

## INTRODUCTION

Heparanase is the only known mammalian endoglycosidase enzyme capable of cleaving heparan sulfate (HS) side chains of HS proteoglycans (HSPG) [1]. HS cleavage results in remodeling of the extracellular matrix (ECM) and basement membrane that underlies epithelial and endothelial cells, thus promoting cellular invasion associated with tumor metastasis and angiogenesis [1]. In addition, heparanase activity releases a variety of HS-bound molecules including growth factors, cytokines, and enzymes that are sequestered in the ECM as a reservoir, thereby ensuring rapid response of cells and tissues to local cues [2]. Heparanase expression is augmented in numerous cancers, including carcinomas, sarcomas, and hematologic malignancies [3–5]. Clinically, elevated heparanase levels are most often associated with increased angiogenesis and metastasis, and reduced postoperative survival of cancer patients [5–10]. Likewise, heparanase silencing reduces cellular invasion and tumor growth [11, 12]. Because heparanase promotes tumorigenesis and is typically not expressed in most normal tissues it is regarded as a promising therapeutic target for cancer patients and other diseases [7, 9, 10, 13–15]. The significant progress in understanding heparanase function and structure over the recent years resulted in the development of heparanase inhibitors, some of which (i.e., Roneparstat, Necuparanib, Muparfostat, PG545) are being evaluated in clinical trials [16].

Lung cancer is the leading cause of cancer-related deaths. Non-small cell lung cancer (NSCLCs) accounts for about 85% of lung cancers. Despite recent advances in molecular and immunotherapy-based treatments, lung cancer still has one of the lowest survival rates of all cancers, with a 5-year survival rate of 18% [17, 18]. Previous studies have shown that heparanase upregulation is observed in the majority (90%) of lung carcinoma biopsy specimens, but not in normal lung tissue collected distant from the tumor lesions [19, 20]. Notably, heparanase induction correlated with increased tumor metastasis, and with reduced patient survival [19, 21].

The overarching hypothesis guiding this research is that heparanase is a key regulator of the aggressive phenotype of NSCLC, and the objective of the study was to further elucidate the role of heparanase in lung cancer. To this end, we applied overexpression and heparanase inhibition (PG545, a synthetic fully sulfated tetra saccharide functionalized with a cholestanyl aglycon) [16, 22] strategies and examined pro-tumorigenic properties of lung cancer cells *in vitro* and tumor growth *in vivo*. Given that patient-derived xenografts (PDX) implanted in immunodeficient mice better resemble the original parent tumor, and that drug responses obtained in PDX models appear highly consistent with responses in patients [23], we established a PDX model system for lung cancer in order to bring anti-heparanase treatment closer to the clinic. We report that PG545 treatment was highly effective in PDX models, exerting inhibition of tumor growth in more than 85% of the PDX. PG545 also abolished macroscopic tumor metastasis. Moreover, we show that PG545 was highly effective in PDX that did not respond to conventional chemotherapy (cisplatin), while other PDX tumors responded well to cisplatin and to a lower extent to PG545, further emphasizing the concept and need for personalized medicine.

## RESULTS

### Heparanase overexpression enhances lung cancer progression

**Figure 1:**
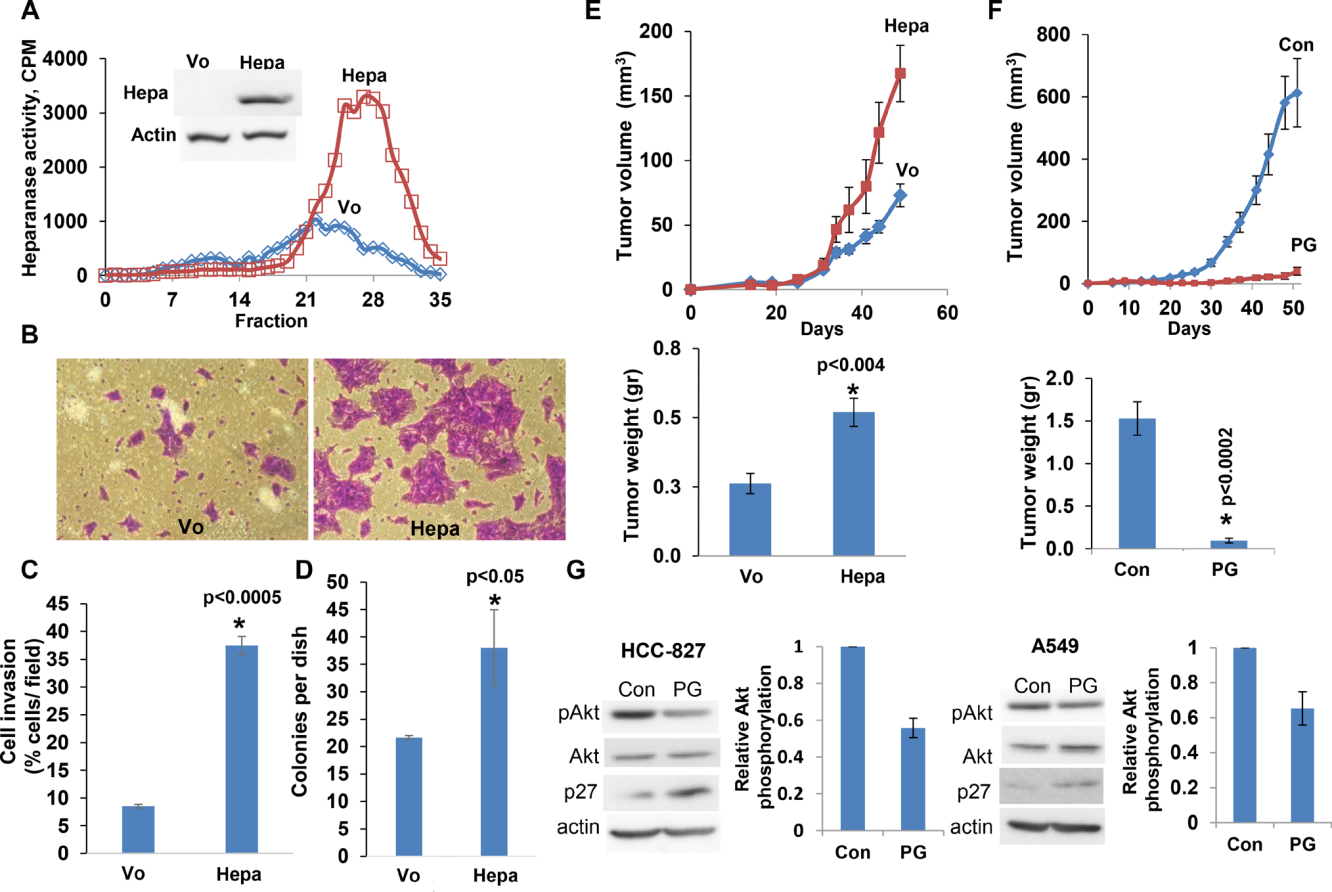
Overexpression of the heparanase gene stimulates lung carcinoma tumorigenesis HCC-827 lung carcinoma cells were stably infected with the heparanase gene (Hepa) and examined for heparanase activity vs mock (Vo) transfected cells (**A**). Cell lysates were incubated with sulfate labeled ECM. Labeled HS degradation fragments released into the incubation medium were subjected to gel filtration on Sepharose 6B column. Inset: immunoblotting for heparanase in Hepa- vs. Vo cells. (**B, C**) Cell invasion. Heparanase (Hepa) and mock (Vo) transfected HCC-827 cells (1 × 10^5^) were plated onto Matrigel-coated 8-μm transwell filters. Invading cells adhering to the lower side of the membrane were visualized (B) and counted (C) after 16 h. (**D**) Colony formation. HCC-827 cells were seeded (2 × 10^3^/35 mm dish) in soft agar and grown for 2 weeks. The number of colonies produced by Hepa vs. control Vo cells was quantified and is shown graphically. (**E, F**) Tumor growth. Heparanase (Hepa) and mock (Vo) transfected HCC-827 cells (2 × 10^6^) were injected subcutaneously to NOD/SCID mice. Tumor volume was calculated from external caliper measurements (E, upper panel). At the end of the experiment on day 50, tumors were resected and weighed (E, lower panel). (**F**) Effect of PG545. HCC-827 cells (2 × 10^6^) were injected subcutaneously to NOD/SCID mice. A group of mice (*n* = 7) was treated with PG545 (20 mg/kg; once a week) and the control group (Con) with vehicle alone. Tumor volume (upper panel) and weight (lower panel) were measured as described above. **G.** Immunoblotting. HCC-827 (left) and A-549 (right) cells (2 × 10^6^) were untreated (Con) or treated with PG545 (PG; 50 mg/ml, 24 h) and subjected to immunoblotting applying anti-phospho Akt (pAkt, upper panels), anti-Akt (second panels), anti-p27 (third panels) and anti-actin (lower panels) antibodies. Note reduced Akt phosphorylation (bar graphs representing densitometric analysis of pAkt relative to total Akt) and up-regulation of p27 in response to PG545.

In order to reveal the significance of heparanase in lung cancer, we first employed lung carcinoma cell lines. Overexpression of heparanase in HCC-827 lung adenocarcinoma cells resulted in a markedly increased heparanase enzymatic activity (release of heparan sulfate degradation fragments from a naturally produced ECM) (Figure 1A) and cellular invasion through a reconstituted ECM (Matrigel; Figure 1B). Quantification of the invasion assay revealed over four-fold increase in invasion capacity of the heparanase overexpressing cells (Hepa) vs. control cells (Vo), differences that are statistically highly significant (*p <* 0.0005; Figure 1C). We next examined the capacity of the cells to grow in soft agar, a characteristic feature that closely reflects the tumorigenic potential of the cells. Heparanase overexpression resulted in a significant increase in the number of cell colonies (37 ± 8 vs. 22 ± 2 for heparanase overexpressing vs. control Vo cells, respectively; Figure 1D, *p <* 0.05). Importantly, heparanase overexpression in HCC-827 lung carcinoma cells enhanced tumor growth by more than 2-fold compared to control (Vo) cells (Figure 1E, *p <* 0.004).

### PG545, a potent heparanase inhibitor, restrains the tumorigenic capacity of lung cancer cells

We next examined the capacity of heparanase inhibitor, PG545, to restrain the tumorigenic capacity of lung carcinoma cells (LLC). We found that tumor cell invasion was profoundly attenuated by PG545, in a dose-dependent manner (Supplementary Figure 1A, 1B; *p <* 0.005). Moreover, the number of colonies developed by A549 cells in soft agar was markedly reduced by PG545 (33 ± 4 vs. 4 ± 2 in control and PG545 treated cells, respectively; *p <* 0.004). Consequently, lung metastasis by LLC cells was noticeably decreased by PG545 (Supplementary Figure 1C). Mechanistically, we found reduced Akt phosphorylation levels in HCC-827 and A549 cells treated with PG545 whereas the expression of p27, an inhibitor of the cell cycle, was increased (Figure 1G). A dose dependent decrease in pAkt was also noted in A549 and HTB-182 lung cancer cells treated with PG545 (Supplementary Figure 1D, 1E). Thus, PG545 apparently decreases cell survival (pAkt) and cell proliferation (p27) pathways, leading to tumor restrain. Strikingly, the growth of tumor xenografts produced by HCC-827 (Figure 1F, *p <* 0.0002), HTB-182 and A549 (Supplementary Figure 2A, B; *p <* 0.0001 and *p <* 0.003, respectively) cells was halted (Figure 1F) or reduced 3–4 fold by PG545, altogether implying that heparanase promotes lung cancer progression and that heparanase inhibitors can be applied to attenuate tumor growth.

### PG545 attenuates the growth of patient derived lung cancer xenografts

**Figure 2:**
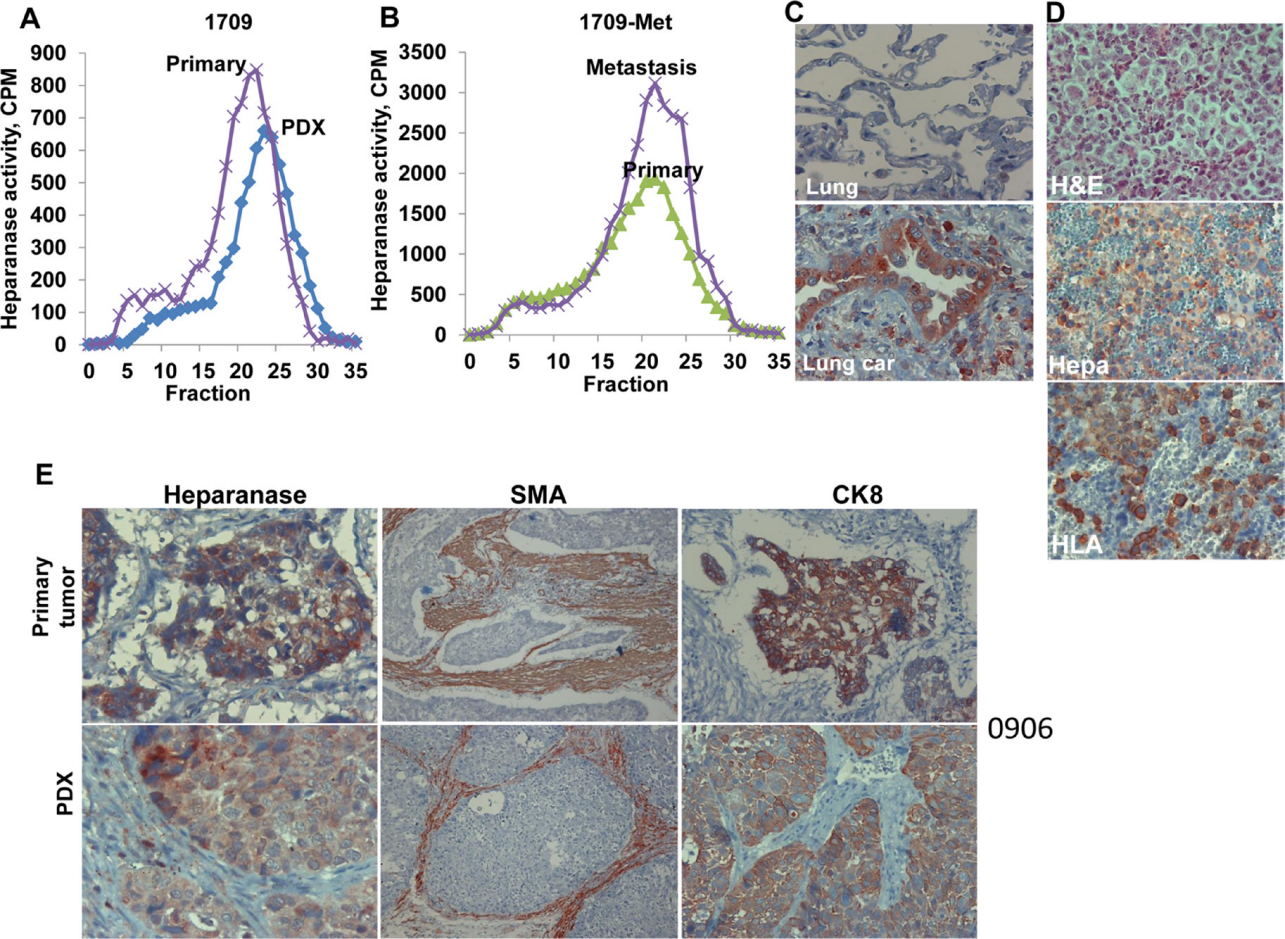
Patient derived xenografts (PDX) maintain characteristics of the respective primary tumors (**A, B** ) Heparanase activity. Portions of the parent (primary) lung tumor biopsy specimen (# 1709) and the respective PDX (**A**), and of the primary PDX and resulting metastasis (**B**) were extracted, homogenized, and examined for heparanase enzymatic activity as described in ‘Methods’. The other portion was fixed subjected to immunostaining for heparanase (Hepa; **C**). Note heparanase immuno-reactivity in the lung tumor (C, lower panel) but not in adjacent normal lung tissue (C, upper panel). Original magnifications: ×100. (**D**) Lymph-node metastasis of PDX 1709 was stained with hematoxylin & eosin (upper panel), anti-heparanase antibody (Hepa, middle panel), and anti-HLA (lower panel) to clearly label human cells within the mouse lymph node. Original magnifications: ×100. (**E**) Sections of the primary 0906 tumor and the resulting PDX were subjected to immunostaining applying anti-heparanase (left), anti-smooth muscle actin (SMA; middle) and anti-cytokeratin 8 (CK8; right) antibodies. A similar expression pattern of heparanase, SMA and CK8 was noted in the parent primary tumors and the respective PDX. Magnification: left and right panels ×100; middle panels ×25.

In order to investigate the utility of anti-heparanase treatments in a lung cancer setting, we established a patient derived xenograft (PDX) model of lung cancer. Fifteen patients with lung cancer (8 adenocarcinoma, 7 squamous cell carcinomas) (Table 1) were enrolled and signed an informed consent for using a biopsy of their tumor for research. Sixty percent of the biopsies (9/15) developed tumor xenografts that were sufficiently large to be advanced into subsequent experiment(s) (Table 1). Importantly, the primary tumor biopsies exhibited a typical high heparanase enzymatic activity that was maintained in the corresponding PDX (Figure 2A, patient 1709), or increased in the metastatic lesion originating from the PDX (Figure 2B). Heparanase immuno-reactivity was noted in the lung tumor (Figure 2C, lower panel) and lymph-node metastasis (Figure 2D, middle panel) but not in the normal lung tissue adjacent to the primary tumor (Figure 2C, upper panel). The occurrence of lymph node metastases was further confirmed by staining for human HLA that stain human cells within the mouse lymph nodes (Figure 2D, lower panel).

**Table 1:** Clinical description and response of lung cancer patients PDXs to heparanase inhibitor, PG545

Pathology	Patient	Gender, Age	TNM	Grade	PDX	PDX Metastasis	Response*
Adeno Carcinoma	**2210**	**F, 68**	**T1N0M0**	**Well**	**Yes**	**Yes**	**Intermediate**
	**0805**	**F, 62**	**T2N1M0**	**Well**	**Yes**	**No**	**Good**
	**1907**	**F, 65**	**T1N1M0**	**Mod**	**Yes**	**No**	**Intermediate**
	1510	M, 62	T1N0M0	Poor	No		
	0809	M, 66	T2N0M0	Well	No		
	0709A	M, 69	T2N0	Well	No		
	0709B	M, 71	T1N0M0	Well	No		
	2008	M, 69	T1N0	Well	No		
SqCC	**1709**	**M, 76**	**T2N0M0**	**Mod-poor**	**Yes**	**Yes**	**Good**
	**1810**	**M, 54**	**T3N2M1**	**Mod**	**Yes**	**No**	**Poor**
	**0906**	**M, 75**	**T2N0MO**	**Mod**	**Yes**	**No**	**Intermediate**
	**1710**	**F, 74**	**T2N1M0**	**Poor**	**Yes**	**No**	**Intermediate**
	**1310**	**M, 70**	**T2N0MO**	**Mod**	**Yes**	**No**	**Intermediate**
	**0507**	**F, 55**	**T1N0M0**	**Mod-poor**	**Yes**	**No**	**Good**
	1308	M, 67	T1N0M0	Well	No		

^*^Response rates: Good >80%

Intermediate 50–79%

Poor <50%

Pathological examination of hematoxylin and eosin (H&E) staining revealed that the PDX tumors maintained the main morphological features of the original primary tumor (Supplementary Figure 3). Immunostaining showed comparable patterns of heparanase expression, recruitment of stromal smooth-muscle actin (SMA)-positive cells, and staining for cytokeratin 8 (CK8) in the PDX and the respective tumor of origin (Figure 2E, patient # 0906 & Supplementary Figure 4).

**Figure 3:**
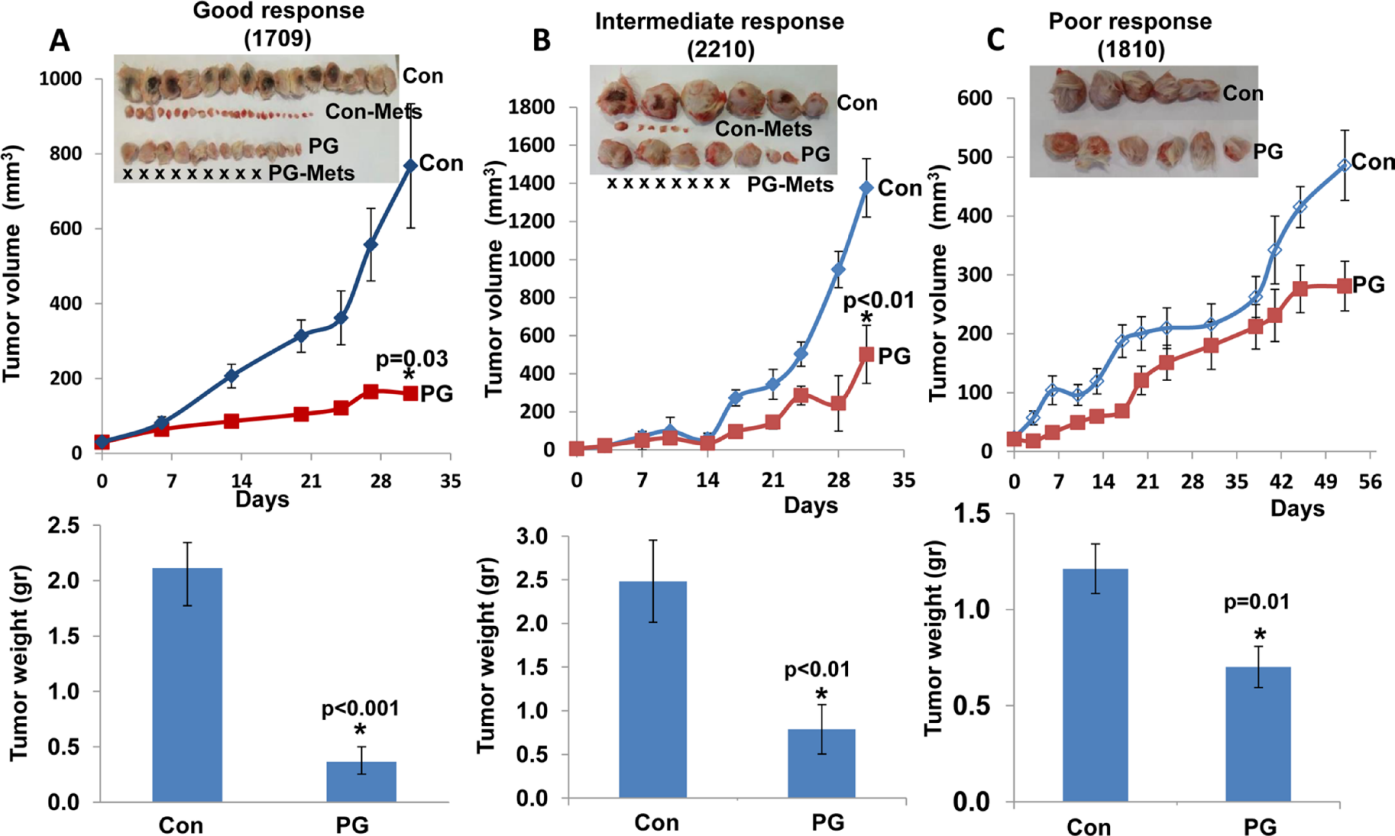
PG545 attenuates the growth of patient derived lung cancer xenografts to various degrees Fresh tumor biopsies were collected from surgery, cut into small pieces and implanted (s.c) into the flank of NOD/SCID mice. Once tumors reached 1–1.5 cm in diameter, tumors were excised, cut into small fragments and re-implanted into NOD/SCID mice. When the tumors became palpable, mice were divided into control (Con) untreated group and study group (PG) that was treated with PG545 (20 mg/kg, once a week). Tumor volume was calculated from external caliper measurements (upper panels). At the end of the experiment, tumors were resected, photographed (insets) along with macroscopic lymph node metastases (Con Mets, PG Mets), and weighed (lower panels). Tumor growth inhibition was categorized as ‘good’ response (**A**, 1709), intermediate response (**B**; 2210), or poor response (**C**, 1810), representing more than 80%, 50–79%, or less than 50% inhibition, respectively. Notably, when spontaneous metastasis to lymph nodes was observed, PG545 not only inhibited tumor growth but also practically prevented tumor metastasis (A, B, insets). X represents no detectable metastases in the indicated treatment regimen.

**Figure 4:**
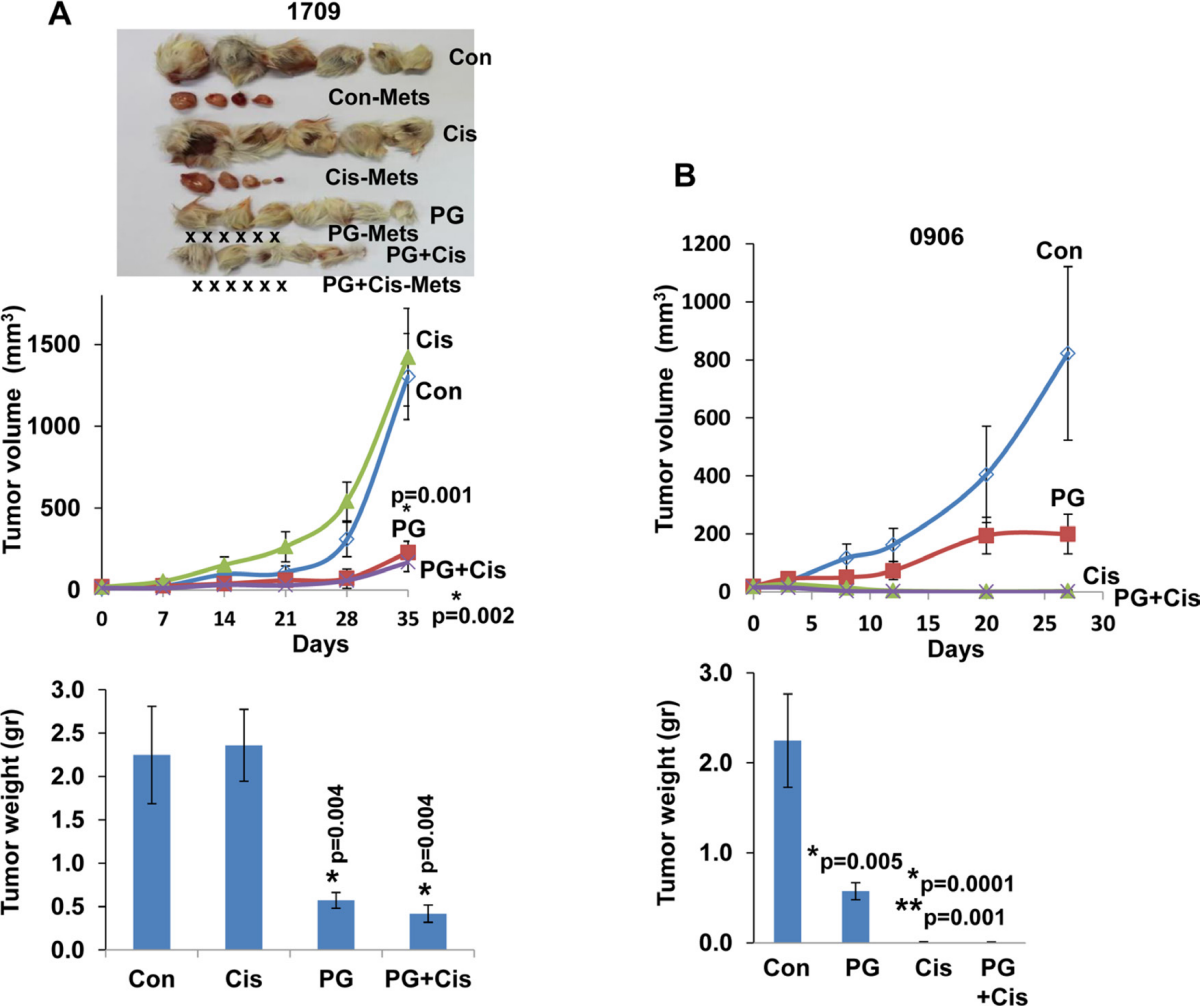
PG545 and cisplatin differentially attenuate the growth of patient derived lung cancer xenografts Fresh patient derived tumor biopsies were cut and implanted (s.c) into the flank of NOD/SCID mice. Once tumors reached 1–1.5 cm in diameter, tumors were excised, cut and re-implanted into NOD/SCID mice. When the tumors became palpable mice were divided into control (Con) untreated group and study groups that were treated with PG545 (PG, 20 mg/kg, once a week), cisplatin (Cis, 3 mg/kg, every two weeks) or both. Tumor volume was calculated from external caliper measurements (upper panels). At the end of the experiment, tumors were resected, photographed along with macroscopic metastases (Con-Mets, Cis-Mets, PG-Mets), and weighed (lower panels). PDX #1709 responded well to PG545 in term of inhibiting tumor growth and tumor metastasis (X represents no detectable metastases in the indicated treatment regimen), but did not respond to cisplatin (**A**), whereas PDX 0906 responded well to cisplatin but only partially to PG545 (**B**). ^*^= *p* values vs Control; ^**^= *p* value vs PG545, for both Cis and PG+Cis.

Interestingly, success rates were significantly higher for squamous cell carcinomas (85%; 6/7) vs. adenocarcinomas (37%; 3/8) (Table 1). We next examined the capacity of PG545 to reduce the growth of the lung PDX measuring both the tumor size as a function of time (Figure 3, upper panels), and tumor weight after its removal at the end of the experiment (Figure 3, lower panels). Mice were administrated with PG545 (20 mg/kg, once a week) once tumors became palpable and tumor growth was inspected. Remarkably, PG545 treatment resulted in reduced tumor growth in 89% of the PDXs (8/9; Table 1, Figures 3, 4); Tumor growth inhibition was categorized as ‘good’ response (Figure 3A) that appeared clinically relevant in 33% (3/9) of the PDXs; intermediate response (Figure 3B) was noted in 45% (5/9) of the PDXs; and only one PDX (11%; # 1810) showed a modest response to PG545 (Figure 3C, poor response; Table 1).

**Figure 5:**
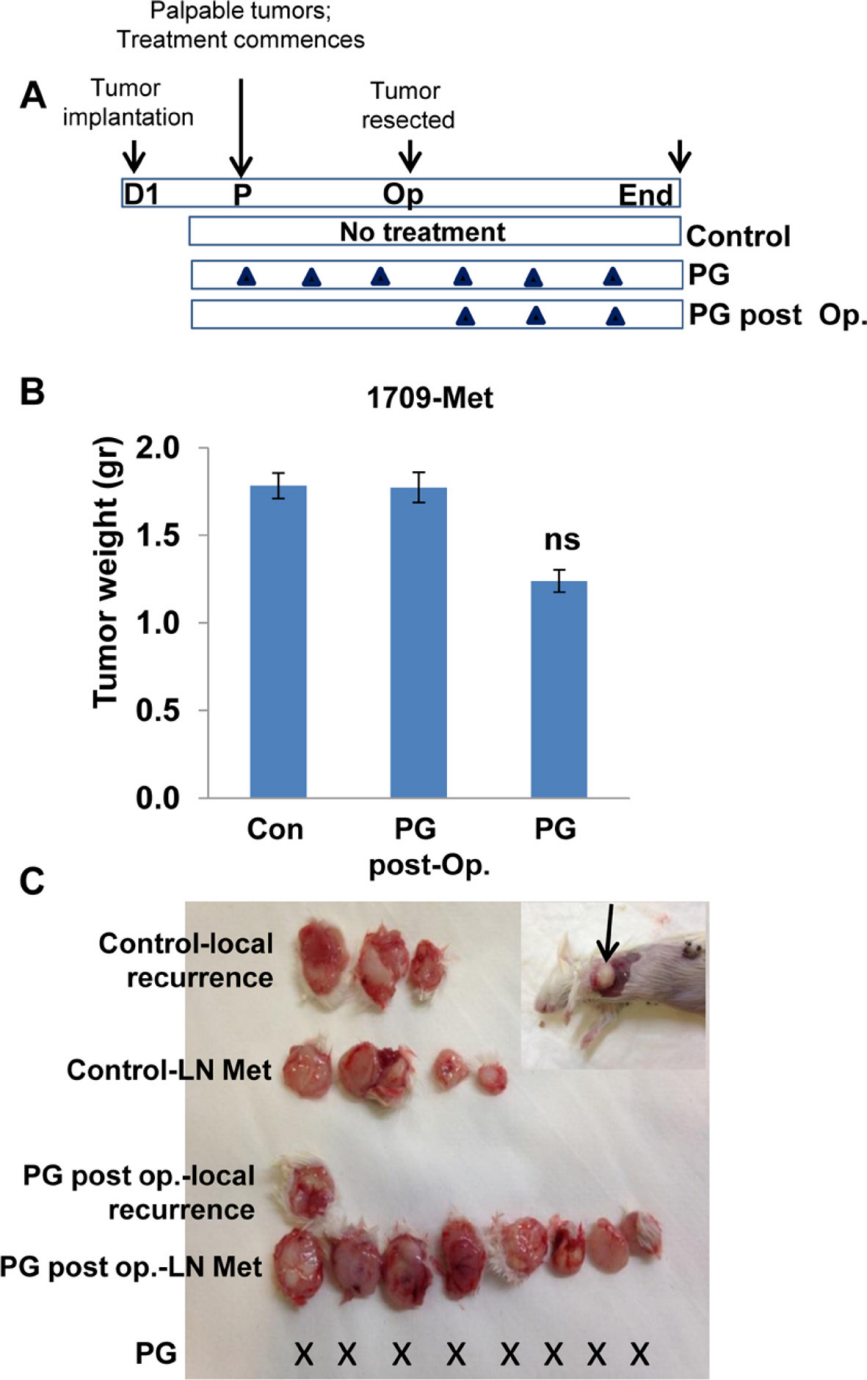
PG545 as neoadjuvant (**A**) Schematic diagram of the experiment. PDX was established from a spontaneous metastasis of PDX 1709 to lymph node (1709-Met). The 1709-Met PDX was implanted in 18 NOD/SCID (D1). Once palpable (day 7), mice were left untreated as control (*n* = 12) or were treated with PG545 (*n* = 6; 20 mg/kg once a week). Three weeks later, when tumors reached a diameter of 10–15mm, tumors were resected and weighed (**B**). Six (out of 12) of the former control mice received PG545 (PG post-Op) while the other 6 mice were kept untreated (Con). One month later, mice were sacrificed and examined for local recurrence and lymph node (LN) metastases (**C**). X represents no detectable tumors or tumor metastases in the indicated treatment regimen. An example of lymph node metastasis in this model is shown in (C, inset). Note that unlike the original 1709 PDX, the 1709-Met PDX does not respond well to PG545 (B). However, PG545 is highly effective in prevention of tumor recurrence and metastasis if present before tumor resection (neoadjuvant).

Spontaneous metastasis to lymph nodes was observed in two cases (#2210 & 1709, Table 1; Figure 3A, 3B and Figure 4A). In these PDXs, PG545 not only inhibited tumor growth but also practically prevented tumor metastasis (Figure 3A, 3B; Figure 4A), thus clearly supporting the pro-metastatic function of the heparanase enzyme. Subsequently, we compared the potency of PG545 (20 mg/kg, once a week) to that of cisplatin (3 mg/kg, every two weeks), a common chemotherapeutic being applied clinically. We found that PDX derived from different patients responded differently to PG545 and cisplatin. Thus, PDX 1709 responded very well to PG545 in term of inhibiting tumor growth and tumor metastasis (‘Good response’; Table 1), but did not respond to cisplatin at all (Figure 4A), whereas PDX 0906 responded very well to cisplatin (*p =* 0.001) but only partially (‘Intermediate response’; Table 1) to PG545 (Figure 4B). Importantly, spontaneous metastasis to lymph nodes by PDX 1709 was evidently prevented by PG545 whereas cisplatin had no effect on tumor growth or tumor metastasis in this patient (Figure 4A). These results reflect the variability among patients and suggest that PG545 can potentially be applied in lung cancer patients in a personalized manner in cases where conventional chemotherapy (i.e., cisplatin) fails. We subsequently examined a lesion taken from a lymph node metastasis of PDX 1709 (1709-Met) for its sensitivity to PG545. Surprisingly, whereas the primary 1709 PDX responded very well to PG545 (Figure 3A; Table 1), the metastasis PDX responded poorly (1709-Met; Figure 5B). We next examined PG545 in a setting of neoadjuvant/adjuvant treatment. In this setting, 1709-Met PDXs were removed once reaching a tumor diameter of ∼10–15 mm, and PG545 was administrated throughout the entire experiment duration (i.e., before and after tumor removal) or only after tumor removal (Figure 5A). Control mice did not receive PG545 al all. Control mice and mice receiving PG545 only after surgery (PG post Op; adjuvant treatment) exhibited local disease recurrence and tumor metastasis (Figure 5C, lower panel). In striking contrast, mice receiving PG545 throughout the entire experiment (neoadjuvant) had no local disease recurrence or tumor metastasis (Figure 5C, lower panel), thus supporting the use of PG545 as a neoadjuvant treatment.

**Figure 6:**
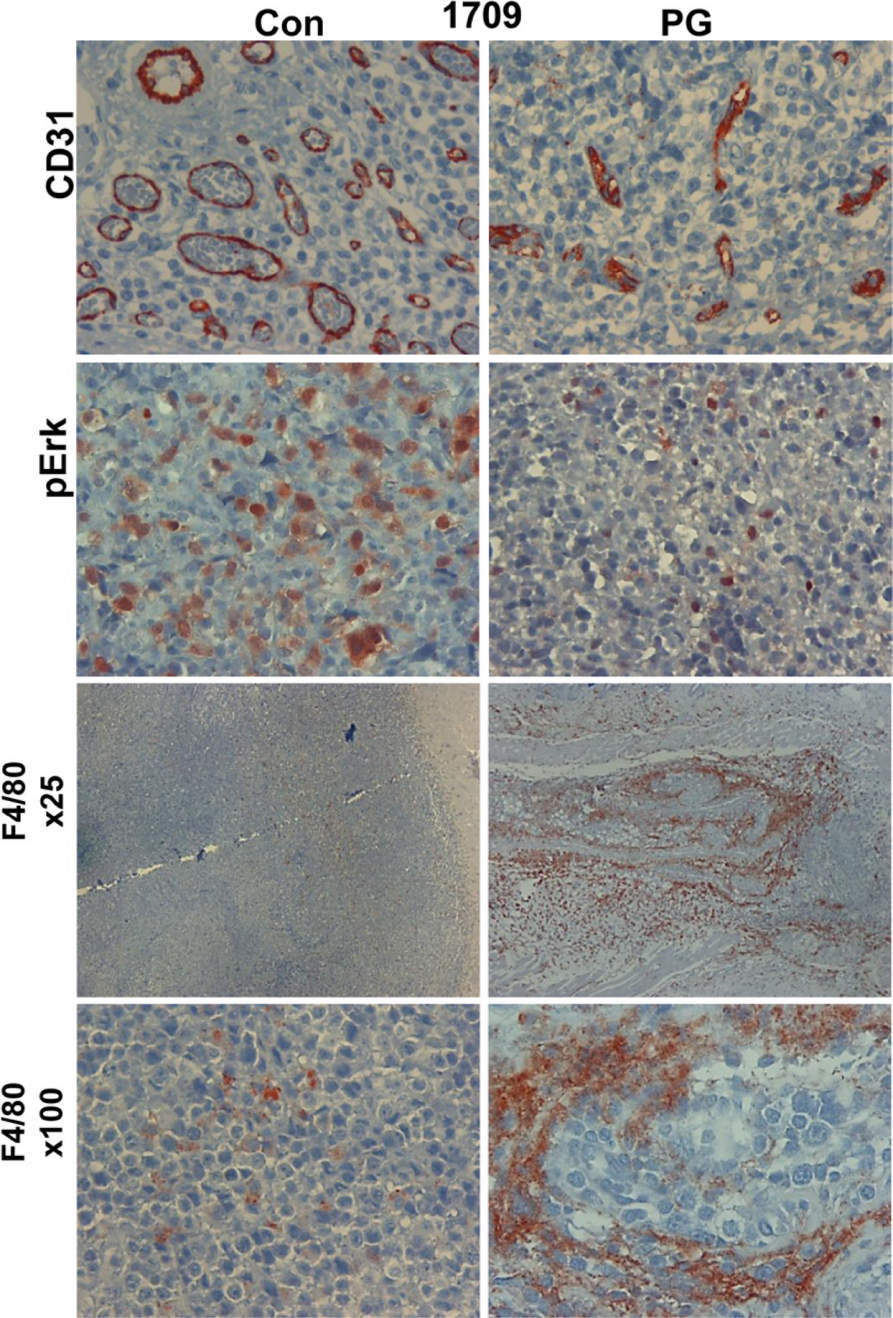
PG545 affects tumor and stromal cells PDX 1709 tumors were treated with vehicle (Con) or PG545 (PG, 20 mg/kg, once a week). At the end of the experiment, tumors were resected and subjected to histological examination and immunostaining with antibodies directed to CD31 (a marker for vascular endothelial cells, upper panels), phospho Erk (pErk; second panels) and against F4/80 (a marker of macrophages; lower panels). Magnification: ×100, except the third panels (F4/80): ×25.

Mechanistically, we found that tumor growth inhibition by PG545 was associated with a decrease in blood vessels density (Figure 6, upper panels). Moreover, blood vessels in PG545-treated tumors appeared collapsed and to a large extent lacked a patent lumen (Figure 6, upper panels), further decreasing their functionality. This is in agreement with the pro-angiogenic feature of heparanase [24, 25] and the anti-angiogenic properties of PG545 [22]. In addition, PG545 resulted in decreased levels of Erk phosphorylation (Figure 6, second panels) and accumulation of macrophages in the tumor periphery (Figure 6, lower panels), resembling localization of macrophages in tumors developed in heparanase-knockout mice [26]. Inhibition of macrophage infiltration by PG545 was observed in other models as well [27, 28]. These results support the use of heparanase inhibitors in lung cancer in a personalized manner, uniquely attenuating tumor growth and metastasis.

## DISCUSSION

Numerous clinical studies have consistently demonstrated that upregulation of heparanase expression correlates with increased tumor size, tumor angiogenesis, enhanced metastasis and poor prognosis [1, 4, 5, 8–10, 15]. In contrast, knockdown of heparanase or treatment of tumor-bearing mice with heparanase-inhibiting compounds, markedly attenuated tumor progression [4, 7, 9, 10, 15], further underscoring the potential of anti-heparanase therapy for multiple types of cancer. Heparan sulfate-mimetics [(i.e., Muparfostat, Roneparstat, Necuparanib, PG545 (= Pixatimod)] that inhibit heparanase enzymatic activity are being evaluated in clinical trials for various types of cancer and appear to be well tolerated [16]. Heparanase neutralizing monoclonal antibodies are being examined in pre-clinical studies [25], and heparanase-inhibiting small molecules are being developed, based on the recently resolved crystal structure of the heparanase protein [29–31]. Of note, PG545 was designed to simultaneously act as both a heparanase inhibitor and an inhibitor of a range of heparan sulfate-binding growth factors that promote angiogenesis [22]. Thus, heparanase inhibition may not be the only reason for the potent anti-tumor activity of PG545, whereas reduced tumor metastasis (Figures 3–5) is likely to result solely from heparanase inhibition.

The present study reveals the significance of heparanase in the pathogenesis of lung cancer. Applying established lung cancer cell lines, we first demonstrated that heparanase overexpression resulted in more aggressive tumor capacities both *in vitro* (cell invasion, anchorage-independent growth) and *in vivo* (xenograft formation, tumor growth and metastasis) (Figure 1). The significance of heparanase in lung cancer progression was further demonstrated by a profound tumor attenuation obtained in response to the heparanase inhibitor PG545 (Figure 1F; Supplementary Figures 1C, 2). To bring anti-heparanase treatment modalities closer to the clinic, we established a lung cancer PDX model which better resembles the behavior and drug responsiveness of a given cancer patient, and more accurately reflects the clinical heterogeneity of the disease than cell lines [23, 32, 33]. Moreover, the large stromal component of tumors is often retained in the PDX models (Figure 2E and Supplementary Figure 4), enabling functional studies targeting the original tumor stroma, thus overcoming another limitation of cell line models [23].

We first demonstrated that the PDX tumors resembled the parent primary lung tumors in their morphology (Supplementary Figure 3), high heparanase enzymatic activity and expression pattern of aSMA, cytokeratin-8 and heparanase (Figure 2; Supplementary Figure 4). Notably, we found that PG545 was highly effective, inhibiting tumor growth in more than 85% of the lung cancer PDX (Table 1), and reaching clinically-relevant inhibition in 33% (3/9) of them as a single agent (good response; Table 1, Figure 3A). Tumor growth inhibition by PG545 was accompanied with impaired angiogenesis evident by collapsed blood vessels and a marked decrease in vascular density (Figure 6, upper panels). This is in agreement with the strong pro-angiogenic properties of heparanase [24, 25, 34], and anti-angiogenic function of PG545 [22]. In addition, treatment with PG545 was associated with reduced phosphorylation levels of Erk (Figure 6, second panel), a most important determinant in non-small cell lung cancer [35], and in agreement with a connection between heparanase and Erk phosphorylation reported in other settings [26, 36–41]. Moreover, macrophages appeared to accumulate at the tumor periphery following PG545 treatment rather than populating the entire tumor mass (Figure 6, lower panels). A similar phenotype was observed in a model of Lewis lung carcinoma cells implanted in heparanase-knockout vs wild type mice [26]. Failure to populate the tumor may contribute to reduced tumor growth given the pro-angiogenic and pro-tumorigenic properties of tumor-resident macrophages [42, 43].

Importantly, we found that PG545 is more effective than cisplatin in certain cases, and vice versa. For example, PDX derived from patient 1709 responded very well to PG545 in term of inhibiting tumor growth and tumor metastasis, but did not respond to cisplatin at all (Figure 4A), whereas PDX from patient 0906 responded very well to cisplatin but only partially to PG545 (Figure 4B), critically stressing the power of personalized medicine. Importantly, PG545 not only attenuated tumor growth but practically prevented spontaneous metastasis of the PDX tumors whereas cisplatin had no such effect (Figure 3A, 3B and Figure 4). This result not only confirms compelling evidence that ties heparanase activity with tumor metastasis [5, 8–10, 15, 44], but also highlights the advantage of PG545 over conventional therapy in combining inhibition of tumor growth and metastasis in a setting most relevant to human patients. Even more dramatic was the inhibition by PG545 of tumor metastasis following removal of the PDX. In this scenario, administration of PG545 prior to, but not after, the removal of the primary tumors practically prevented metastasis (Figure 5C), thus supporting the use of this compound as a neoadjuvant treatment. Administration of PG545 after removal of the tumor appears too late to suppress the proliferation of metastatic cells that have already disseminated and settled in the lymph-nodes. Moreover, removal of the primary tumor has been previously shown to stimulate the development and growth of dormant metastases [45] thereby limiting the effectiveness of PG545 administered post-operation. In contrast, it is conceivable that in mice receiving PG545 throughout the entire experiment metastatic dissemination is inhibited by the drug to start with, providing a possible explanation for the high efficacy observed when the drug is administrated prior to resecting the primary tumor. Presently, all the patients enrolled in our study are still alive and hence it is too early to conclude the outcome of the patients after 12-18 months of follow-up. Also, PG545 just ended phase I clinical trial and cannot be given to lung cancer patients which still relay on conventional chemotherapy (i.e. cisplatin). It is hoped, nonetheless, that our results will pave the way for testing the efficacy of PG545, alone and in combination with other treatments (i.e., chemotherapy), in more advanced clinical trials. Importantly, PG545 was found to enhance the anticancer activity of chemotherapies in animal models of ovarian and pancreatic cancer [46, 47], providing the basis for an ongoing phase Ib clinical trial of PG545 in combination with nivolumab in patients with advanced solid tumors and in patients with metastatic pancreatic cancer (Zucero Therapeutics, Austyralia). Likewise, the heparanase inhibitor Roneparstat (formerly SST0001) in combination with a proteasome inhibitor (bortezomid) or melphalan was found to be an effective therapeutic strategy to inhibit disseminated tumor growth and overcome drug resistance in myeloma bearing mice [38].

Taken together, our results suggest that PG545 can be applied for lung cancer therapy in a personalized manner where conventional chemotherapy fails, thus bringing anti-heparanase treatment modalities closer to the clinic.

## MATERIALS AND METHODS

### Patients

The study included 15 patients with lung cancer (8 adenocarcinoma, 7 squamous cell carcinomas) (Table 1) who were diagnosed in the Department of Thoracic Surgery (Rambam Health Care Campus, Haifa, Israel) and enrolled from August 2015 to October 2016. Surgical specimens were taken before any treatment. Patients signed an informed consent for using a biopsy of their tumor for research. The study protocol was approved by the Institutional Review Board. The clinical data from all patients were reviewed, and patients were restaged according to the 2006 American Joint Committee on Cancer staging system.

### PDX model system

Tumor samples were obtained directly from the operating room, dissected carefully, trimmed into small pieces (approximately 2–3 mm) under sterile conditions and implanted subcutaneously (s.c; one tumor fragment per mouse) into the flank of 5-7 NOD/SCID mice within 3 h after collecting the tumor sample from the patient. PDX development was assessed by palpation of the site of implantation [23, 48]. Once tumors reached 1–1.5 cm in diameter, mice were euthanized and tumors were excised and cut into small fragments (2–3 mm) that were re-implanted (one tumor fragment per mouse) into 16-20 SCID mice. When the tumors became palpable (typically 10–20 days post cell inoculation), mice were divided into study groups that included a control untreated group and 1–3 study groups that were treated with PG545 (20 mg/kg, intraperitoneal injection of 0.1 ml/mouse, once a week) [22], cisplatin (intraperitoneal injection of 0.1 ml/mouse, 3 mg/kg, every two weeks) or both. Drugs were solubilized in saline. Tumor size was inspected over time by externally measuring the tumor in 2 dimensions using a caliper. At termination, tumor xenografts and lymph-node metastases were removed, weighed, and subjected to histological examination, and determination of heparanase level and enzymatic activity. All animal experiments were approved by the Animal Care Committee of the Technion (Haifa, Israel). The response to treatment was defined as good, intermediate or poor upon tumor growth inhibition by more than 80%, 50–79%, or less than 50%, respectively. The study was approved by the Helsinki Committee of the Rambam Health Care Campus.

### Antibodies and reagents

Anti-heparanase rabbit polyclonal antibody (#733) was prepared against a synthetic peptide corresponding to the enzyme substrate binding domain [49] and was used for immunostaining. Anti-Akt, anti-Erk, anti-phospho Erk, and anti-p27 antibodies were purchased from Santa Cruz Biotechnology (Santa Cruz, CA); anti-phospho-Akt antibody was purchased from Cell Signaling Technologies (Beverly, MA). Anti-actin and anti-smooth muscle actin (SMA) monoclonal antibodies were purchased from Sigma (St. Louis, MO). Rat anti-mouse CD31 antibody was from Dianova (Hamburg, Germany); anti-HLA and anti-cytokeratin 8 (CK8) antibodies were purchased from Abcam (Cambridge, MA). Rat anti-mouse F4/80 antibody was purchased from Serotec (Oxford, UK). PG545 (= pixatimod), was kindly provided by Zucero Therapeutics, Brisbane, Queensland, Australia.

### Cells, cell culture, cell invasion and colony formation

Human [A549, HCC-827 (adenocarcinomas), and HTB-182 (squamous cell carcinoma)] and a mouse (Lewis lung carcinoma) lung cancer cell lines were purchased from the American Type Culture Collection (ATCC). Cells were cultured in DMEM medium (Biological Industries, Beit Haemek, Israel) supplemented with 10% fetal calf serum (FCS) and antibiotics. Cells were infected with a lentivirus carrying the human heparanase cDNA, selected with blasticidin and pooled, as described [50]. Colony formation in soft agar and Matrigel invasion assay were performed essentially as described [50, 51]. To quantify cellular invasion, the area covered by invading cells/filed was computed and presented graphically. Immunoblotting and ECM degradation heparanase assay were carried out as described [25–27, 50, 52, 53].

### Histology and immunohistochemistry

Immunostaining of formalin-fixed, paraffin-embedded tumor xenograft sections was carried out essentially as described [26, 27, 53]. Images were acquired by a Nikon ECLIPSE microscope and Digital Sight Camera (Nikon, NY, USA).

### Tumor xenografts

Control (Vo) and heparanase overexpressing HCC-827 and HTB-182 cells (5 × 10^6^/100 μl) were injected subcutaneously (s.c) into the right flank of NOD/SCID mice and tumor growth was inspected over time by externally measuring the tumor in 2 dimensions using a caliper. Cells were similarly implanted subcutaneously and mice were treated with PG545 (intraperitoneal, 20 mg/kg, once a week) starting when the tumor became palpable. Control mice received PBS. At termination, tumor xenografts were removed, weighed, and subjected to histological examination and determination of heparanase level and activity.

### Statistics

Data are presented as mean values ± SD. Statistical significance was analyzed by 2-tailed Student’s *t* test. Values of *P* < 0.05 were considered significant. Data sets passed D’Agostino-Pearson normality (GraphPad Prism 5 utility software).

## SUPPLEMENTARY MATERIALS FIGURES


