# Supplementary material for: Patient derived xenografts (PDX) predict an effective heparanase-based therapy for lung cancer
Source: Oncotarget. 2018 Apr 10;9(27):19294–306. doi: 10.18632/oncotarget.25022 (PMC5922397; doi:10.18632/oncotarget.25022)
Supplement: Supplementary file 1 [file oncotarget-09-19294-s001.pdf]

Patient derived xenografts (PDX) predict an effective heparanase-based therapy for lung cancer

SUPPLEMENTARY MATERIALS

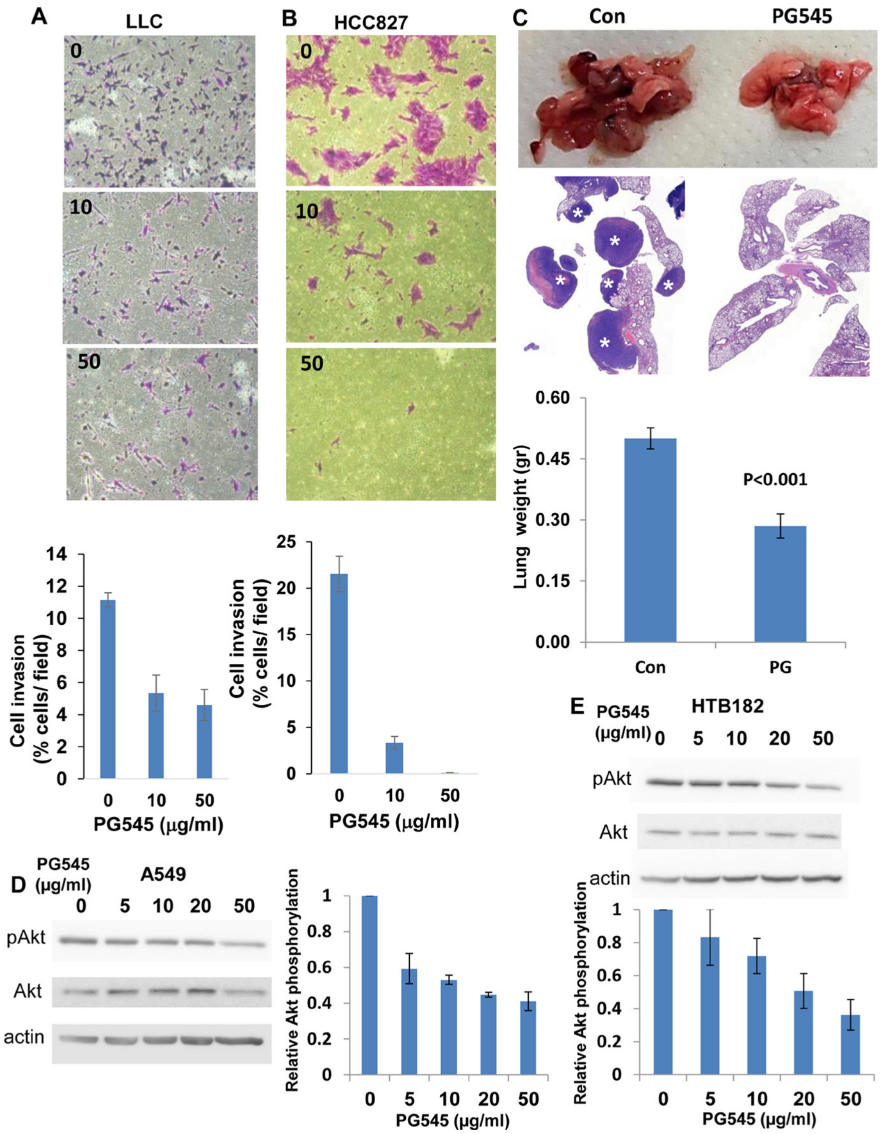

Supplementary Figure 1: Effect of PG545 on lung carcinoma cell invasion, Akt phosphorylation and tumor growth. (**A, B**) Cell invasion. Mouse Lewis lung carcinoma (LLC) (A) and human HCC-827 (B) cells (1×10^5) were plated onto Matrigel-coated 8-μm transwell filters without (0) or with 10 or 50 mg/ml PG545. Invading cells adhering to the lower side of the membrane were visualized (upper panels) and counted (lower panels) after 16 h. (**C**) Tumor metastasis. LLC were injected to the tail vein of C57Bl/6 mice untreated (Con) or treated with PG545 30 min prior to cell inoculation. Lungs were harvested 30 days later, and examined for lung metastases by gross inspection (upper panel) and by histology (second panels). *****denotes lung metastasis. (**D, E**) Immunoblotting. A549 (**D**) and HTB-182 (**E**) human lung carcinoma cells (2×10^6) were left untreated (0) or treated (16 h) with the indicated concentrations PG545 and cell lysates were subjected to immunoblotting applying anti-phospho-Akt (pAkt; upper panels), anti-Akt (second panels), and anti-actin (lower panels) antibodies. Note decreased Akt phosphorylation in response to PG545. Bar graphs (D, right; E, bottom) denote densitometric analysis of pAkt relative to total Akt.

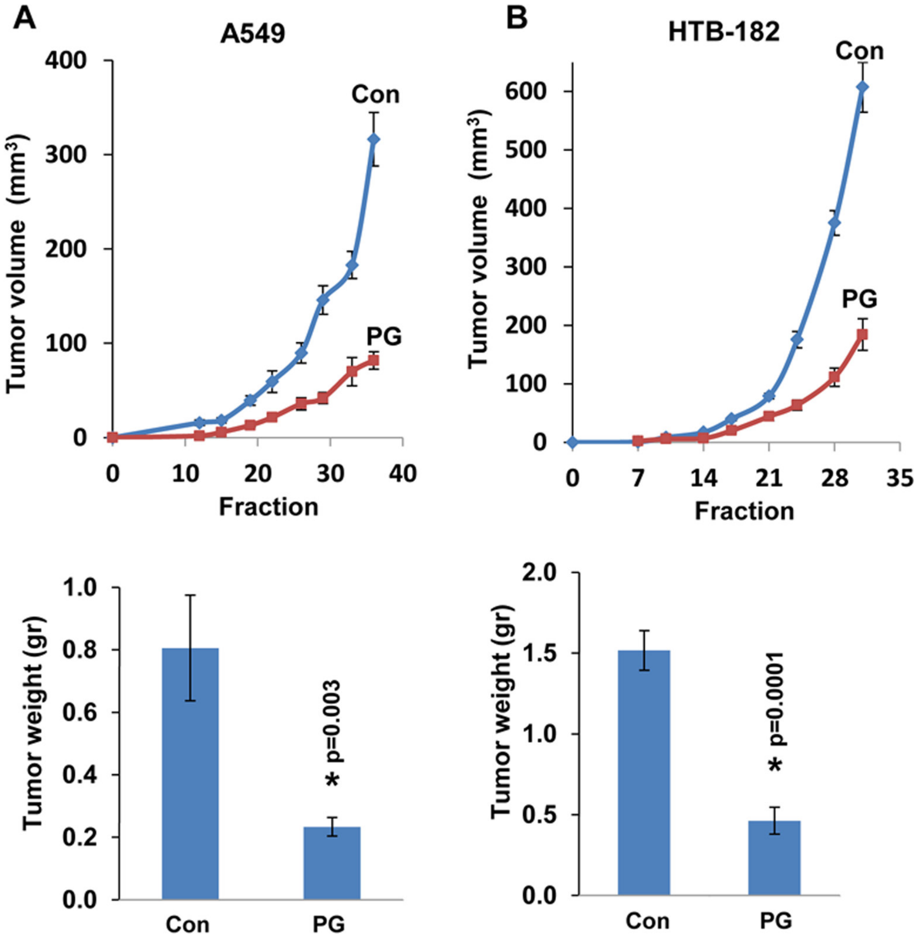

Supplementary Figure 2: (**A, B**) PG545 attenuates the growth of tumor xenografts produced by lung carcinoma cells. A-549 (**A**) and HTB-182 (**B**) cells (5×10^6) were implanted subcutaneously in NOD/SCID mice ($n = 7$) and mice were administrated with vehicle (Con) or treated with PG545 (20 mg/kg; once a week). Tumor volume was calculated from external caliper measurements (upper panel). At the end of the experiment, tumors were resected and weighed (lower panel).

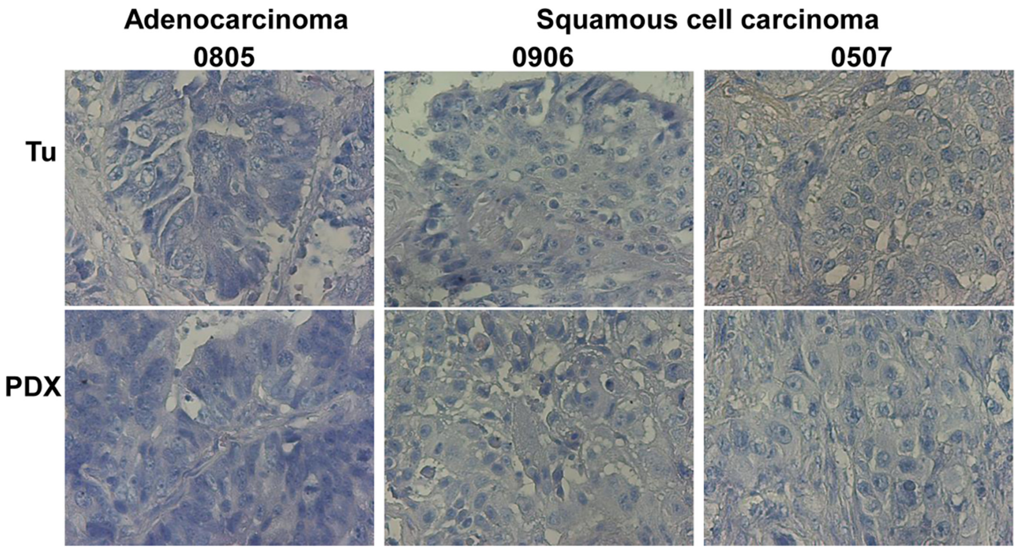

Supplementary Figure 3: Hematoxylin & eosin staining. Five-micron section of the indicated parent (Tu) and the resulting PDX were stained with H&E. Note that the cell morphology is retained in the PDX. Original magnifications ×100.

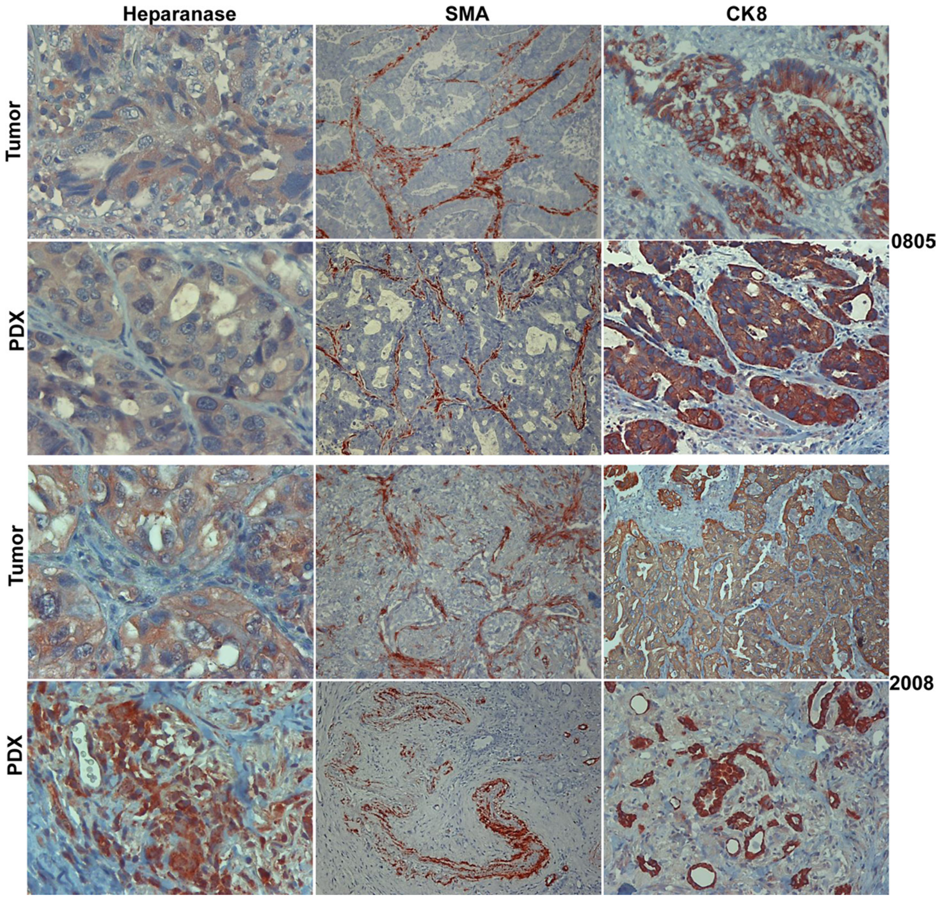

Supplementary Figure 4: Sections of the indicated primary tumor and the resulting PDX were subjected to immunostaining applying anti-heparanase (left), anti-smooth muscle actin (SMA; middle) and anti-cytokeratin 8 (CK8; right) antibodies. A similar expression pattern of heparanase, SMA and CK8 was noted in the parent primary tumors and the respective PDX. Magnification: left and right panels ×100; middle panels ×25.
